# Tuning of Human Modulation Filters Is Carrier-Frequency Dependent

**DOI:** 10.1371/journal.pone.0073590

**Published:** 2013-08-29

**Authors:** Andrew J. R. Simpson, Joshua D. Reiss, David McAlpine

**Affiliations:** 1 Centre for Digital Music, Queen Mary University of London, London, United Kingdom; 2 Ear Institute, University of Central, London, London, United Kingdom; Universität Bielefeld, Germany

## Abstract

Recent studies employing speech stimuli to investigate ‘cocktail-party’ listening have focused on entrainment of cortical activity to modulations at syllabic (5 Hz) and phonemic (20 Hz) rates. The data suggest that cortical modulation filters (CMFs) are dependent on the sound-frequency channel in which modulations are conveyed, potentially underpinning a strategy for separating speech from background noise. Here, we characterize modulation filters in human listeners using a novel behavioral method. Within an ‘inverted’ adaptive forced-choice increment detection task, listening level was varied whilst contrast was held constant for ramped increments with effective modulation rates between 0.5 and 33 Hz. Our data suggest that modulation filters are tonotopically organized (i.e., vary along the primary, frequency-organized, dimension). This suggests that the human auditory system is optimized to track rapid (phonemic) modulations at high sound-frequencies and slow (prosodic/syllabic) modulations at low frequencies.

## Introduction

The primary feature represented by the peripheral auditory system is sound frequency; the basilar membrane of the cochlea is arrayed, from base to apex, according to a tonotopic representation, with high frequencies resolved at the basal end and low frequencies at the apical. Tonotopic organization is apparent up to at least primary auditory cortex [1], which has been characterized as showing an intensity-independent representation of sound [2,3] responding predominantly to stimulus contrast. Numerous studies have revealed a preference for “natural” 1/*f* modulation statistics [4,5] in the auditory system [6–8] and this selectivity has been localized to auditory cortex [7,8]. Models comprising central modulation filter-banks have been proposed [9–11], including the existence of independent modulation filters in the human auditory cortex [12]. Presumably, these cortical modulation filters (CMF) represent separate neuronal populations, each with different tuning to modulation rate [13]. Xiang et al. [12] have suggested that, much like the ‘beating’ that occurs within the auditory filters of the cochlea itself, CMFs are nonlinear and produce sum/difference products when two modulations (at different rates) exist within the same filter.

Speech intelligibility has been shown to be dependent on sensitivity to slow temporal modulations [14,15]. Assuming CMFs play a key role in coding speech, particularly in background noise (i.e., ‘cocktail-party’ listening [13,16,17]), a potential strategy for separating speech from background noise, and one recently suggested by Ding and Simon [13], is that CMFs are carrier-frequency dependent, i.e., the modulation rate to which CMFs are tuned increases systematically along the tonotopic gradient. This would allow speech signals of a given pitch (here, carrier frequency) to maximize their energy ratio, within a given CMF, against that of background noise or competing speech at different pitch. This strategy also makes sense from the perspective of the Nyquist-Shannon rate-limits imposed by peripheral auditory filters, the bandwidths of which increase (in Hertz terms) with increasing centre frequency, making it theoretically possible to convey increasingly higher modulation rates. In support of this, Lakatos et al. [17] demonstrated tonotopically-arranged entrainment of neural activity in the cortex of non-human primates, suggestive of a tonotopic arrangement of CMFs. Further evidence in support of a tonotopic arrangement of CMFs comes from neuroimaging studies (as reviewed by Zarate and Zatorre [18]), where a ‘dual stream model’ of the cortex has been proposed to account for hemispheric spectro-temporal processing differences (for musical stimuli) equivalent to those observed by Lakatos et al. [17]. It follows from this that if CMF tuning is carrier-frequency dependent, it might be the product of tonotopic variation in underlying neuronal physiology.

Since human cortex (like that of the monkey) is tonotopically mapped [1], if CMFs are carrier-frequency dependent, then subcortical spread of excitation across the tonotopic gradient (likely initiated at the level of the basilar membrane) may have an equivalent ‘cortical spread of modulation’ effect, where the peripheral spread of excitation along the tonotopic gradient spreads modulation across nearby CMFs. This spread of modulation might then result in similar level-dependent, nonlinear interactions to those observed by Xiang et al. [12], such that CMF tuning would broaden with increasing sound level to cause ‘simultaneous modulation masking’, much as the peripheral auditory filters cause simultaneous energetic masking [19].

Previous psychoacoustic studies have suggested that intensity discrimination is carrier-frequency dependent - intensity discrimination varies as a function of stimulus duration [20] and as a function of sound level [21–23]. However, these findings have not been systematically verified or related to cortical processing of stimulus contrast. More recent studies have suggested a key role of contrast in detecting changes in sound intensity [24–27]. Here, we investigated modulation filters using a novel behavioral method derived from psychoacoustics. Listeners were asked to detect linearly-ramped increments (i.e., the just noticeable difference [JND]), in pure-tone carriers, at effective modulation rates between 0.5 and 33 Hz. These rates span the range of prosodic (<5 Hz), syllabic (5 Hz) and phonemic (20 Hz) rates commonly found in speech [12,14,15]. By varying the level and frequency of the carrier signal, we characterized the tuning of the modulation filters (in terms of *modulation-rate-sensitivity* and *modulation-depth-sensitivity*) as a function of carrier frequency and level. Our data support the view, as suggested by Ding and Simon [13] and implied by Lakatos et al. [17], that modulation filters are systematically dependent on carrier frequency. Given that the cortex is known to be tonotopically organized [1], this suggests that CMFs are similarly organized, in covariance with the well-established tonotopic map, and in support of the ‘dual stream’ model [18]. We also observe that modulation sensitivity changes as a function of sound level in a manner that may be attributable to spread of excitation across modulation filters as sound level increases. In summary, our data suggest that the human auditory system is optimized to track rapid modulations at high sound-frequencies and slow modulations at low, and supports a model of cortical function based on tonotopically-organized modulation filters.

## Method

**Figure 1:**
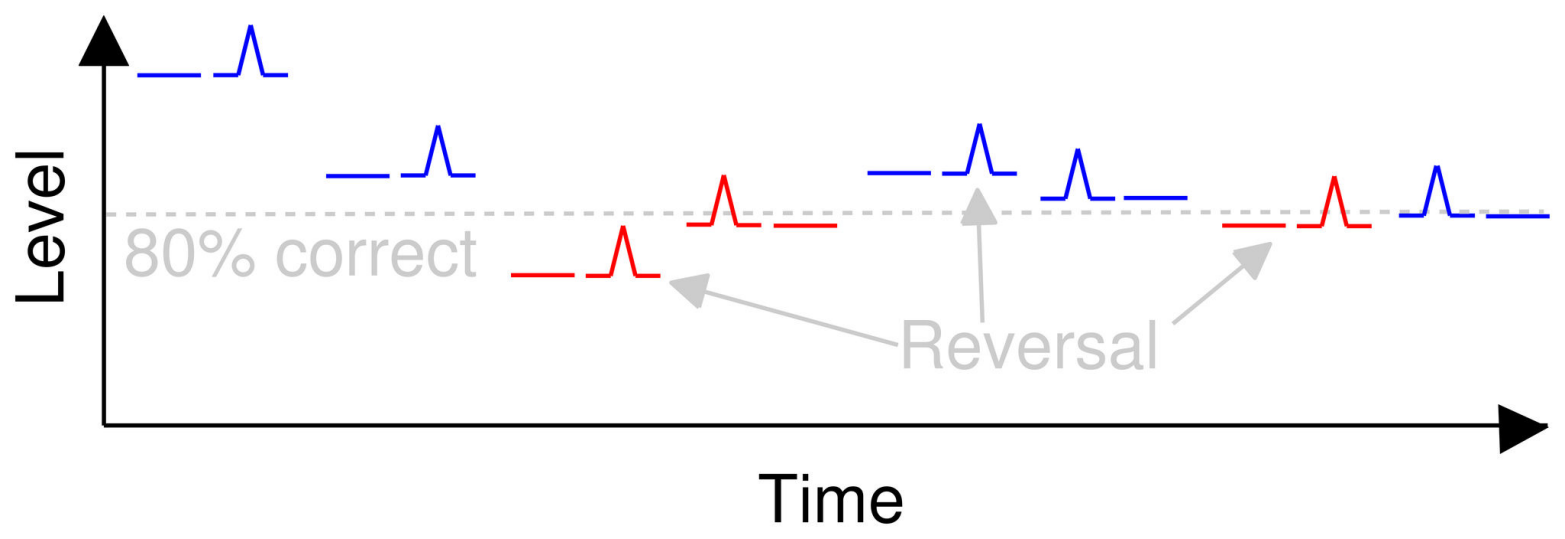
Illustration of the inverted method. Pairs of pure-tones are presented, one of which contains a ramped increment. The ramp size and duration is fixed throughout, whilst listening level is adaptively changed until the procedure converges on 80% correct performance. When the listener correctly identifies the location of the ramped intensity increment the listening level is reduced, otherwise the listening level is increased, depending on a rule (simplified here to a 1-up, 1-down rule). Correct responses (blue) result in decreased listening level and incorrect responses (red) result in increased listening level. The 80% correct threshold level is estimated by averaging the listening level measured at several points where the adaptive procedure changes direction (‘reversals’). The step size of the level change reduced after a reversal and the procedure eventually converges on the 80% correct point.

The prevailing experimental paradigm for assessing the intensity JND specifies a fixed listening level and an adaptively-varied increment size [24–27]. However, due to individual differences in auditory physiology, small changes in listening level produce large changes in the size of the intensity JND (e.g., [28]) and, near threshold, the mapping is both extremely nonlinear and highly individualized. When this method is applied to a medium sample size, even if individual listeners are extremely reliable, the mean results for such a sample constitute a gross averaging (blurring) of subtle trends in the data that potentially characterize modulation filter tuning. In previous studies [21–23], listening levels were fixed relative to the absolute threshold (i.e., sensation level – SL) for each listener in order to provide comparison between intensity JNDs at different carrier frequencies. This resulted in the observation of carrier-frequency dependence in the JND as a function of SL but the findings were not related to temporal integration (see below), a major topic of more recent investigations [24–27]. Here, we invert the traditional experimental paradigm [24–27] such that listening level is adaptively varied and the size of the increment is held constant (see Figure 1). This normalizes between-subject variance caused by individual differences in absolute thresholds.

By assessing JNDs at different ramp durations, a *modulation-rate-sensitivity* function is produced [24–27], characterizing the relative sensitivity of the modulation filter to different ramp (i.e., modulation) rates. From this function, tuning for the modulation filter at each carrier frequency can be estimated. For modulation filters tuned to low modulation rates (e.g., prosodic or syllabic; 5 Hz or less), the *modulation-rate-sensitivity* function will show greatest sensitivity to the slowest ramps (1000 ms). For modulation filters tuned to higher modulation rates (e.g., near phonemic; 20 Hz or more), the *modulation-rate-sensitivity* function will show greatest sensitivity at the higher modulation rates. By testing at different heights of ramp (with a fixed ramp duration of 5 Hz effective modulation rate), *modulation-depth-sensitivity* functions can be produced and level dependence in the modulation filters can be probed. If the tuning of modulation filters varies as a function of carrier frequency, level-dependent trends with carrier-frequency should be observed because for a fixed modulation rate, as carrier frequency is varied some CMF will be operating in the tuned peak and other CMF will be operating in the skirts. Therefore, this also allows us a window into possible spread-of-modulation effects.

### Inverted method

Detection threshold levels were obtained for up-down ramped increment envelopes added to the center of 4-s long pure-tone carrier-signals, for nine listeners. Listeners were presented with pairs of matched 4-s long tones, one of which (at random) contained a linear up-down increment. The listening level was started high, so that the increment was clearly audible, and then varied adaptively until threshold level was determined. If the subject correctly selected the tone with the increment the listening level was reduced, and, if incorrectly, the listening level was increased. Thresholds were estimated by averaging the listening level at several such decision rule points.

By separately varying the frequency of the carrier and the size and duration of the increment envelopes, corresponding equal-JND-level contours were produced and, from these contours, threshold-level functions of ramp duration and of ramp size, i.e., *modulation-rate-sensitivity* and *modulation-depth-sensitivity* functions obtained. Parametric analysis of the data was employed to reveal systematic trends with carrier frequency.

Two experiments were conducted. The first experiment was designed to illustrate the *modulation-rate-sensitivity* tuning of modulation filters as a function of carrier frequency. The second experiment was designed to illustrate the associated *modulation-depth-sensitivity* tuning within the modulation filters for modulations at approximately 5 Hz (i.e., syllabic rate). In the first experiment (the *temporal* experiment), the size of the intensity increment was fixed at 3 dB. Half-ramp duration of the increment was set to either 15, 50, 100 or 1000 ms for each block (equivalent to a modulation rates of 33, 10, 5 or 0.5 Hz respectively). This produced a set of four contours, from which *modulation-rate-sensitivity* functions of increment ramp duration could be extracted. In the second experiment (the *magnitude* experiment), the increment size was set to either 1, 2 or 3 dB for each block, and half-ramp durations of 100 ms (corresponding to a modulation rate of approximately 5 Hz) were used for each respective block. This produced a set of three contours, from which *modulation-depth-sensitivity* functions could be extracted. From here onwards, we refer to the ramp durations of 15, 50, 100 or 1000 ms in terms of the equivalent modulation rates of 33, 10, 5 or 0.5 Hz respectively.

A *post-hoc* analysis was performed quantifying systematic trends in the shapes of the *modulation-rate-sensitivity* and *modulation-depth-sensitivity* functions, and a correlation analysis was employed to assess correlations in the two measures that may be attributable to the properties of the modulation filters.

### Near Miss

A prerequisite of our method is that, for a given increment, detection improves with increases in listening level. Weber’s Law states that the ratio of intensity to the intensity JND should be constant [29] and has been shown to be approximately true for wideband signals [30]. However, in the case of pure tones, Weber’s Law has been shown not to hold (e.g., [28]) and the characteristic steady (monotonic) decrease in the JND with increasing sound level is referred to as the ‘near-miss to Weber’s Law’ [31]. The near miss necessary for our method has been shown to hold for continuous 1-kHz carriers up to 85 dB SPL, corresponding to around 80 dB above threshold [28]. In this study, by using relatively large increments, we limit our investigation to the range between threshold and around 40 dB above threshold. However, it should be noted that non-monotonicity was observed for gated 1-kHz signals above 90 dB SPL in the above-mentioned study [28], and that the near-miss is less well defined in (or even absent from) studies employing noise maskers (e.g., [32]).

### Listeners

Nine unpaid volunteer subjects served as listeners in the experiments. Six male subjects and three female subjects took part. The mean age of the subjects was 27 (min: 21, max: 33). All reported normal hearing and previous experience of participating in listening tests. All participants were naïve concerning the purpose of the test.

#### Ethics Statement

Participants were voluntary, unpaid and gave informed verbal consent before the experiment. Participants were free to withdraw at any point. Tests were run on an ad-hoc basis. Written consent was not deemed necessary due to the low (safe) sound pressure levels employed in the test but the consenting volunteers were documented. The experimental protocol (including consent) was approved by the ethics committee of Queen Mary, University of London.

### Stimuli

Stimuli were generated digitally at 24-bit resolution. A pair of Beyerdynamic DT100 isolating headphones was used to present the stimulus to listeners directly from a computer, at a sampling rate of 44100 Hz. Presentation was diotic (identical in both ears). The carriers were gated on and off using 10-ms raised-cosine ramps. In both experiments, detection threshold levels were obtained at carrier frequencies of 62, 125, 250, 500, 1000, 2000, 4000, 5650, 8000, 11300 and 16000 Hz. Pure-tone (sinusoidal) carriers were presented in blocks of JND = 1, 2 and 3 dB. In this paper, JND is defined as: 10*log*_10_(1+Δ*I*/*I*), *I* = intensity. Carrier frequency was varied inside blocks. Symmetrical ramped envelopes were added to the tone carriers. A ramped envelope for a given duration consisted of a linear increment ramp of that duration, immediately followed by a linear decrement ramp of the same duration. The ramp envelopes were located in the temporal center of the 4-s long carrier. The increment was set to a fixed value within any given block. In the *temporal* experiment, linear up-down ramped increments with effective modulation rates of 0.5, 5, 10 or 33 Hz were imposed upon 4-s pure-tone carriers. Threshold levels were obtained for JND = 3 dB. In the *magnitude* experiment, 5 Hz modulations were used and threshold levels were obtained for JND = 1, 2 and 3 dB.

The range of JNDs was chosen to lie within the known monotonic range. The range was also limited to relatively large values of JND (>=1 dB) for the reason that very small values of JND at low and high carrier frequencies would have required listening levels beyond those possible with the available apparatus.

### Procedure

For each carrier frequency within a block, an adaptive three-down one-up, two-interval forced-choice (2IFC) procedure was employed which estimates the 79.4% correct identification [33]. Each pair of signals that constituted a trial, presented in random order, consisted of one carrier that contained a ramp envelope and a second carrier that contained no ramp. The signal pairs were presented with silent inter-signal intervals of 0.5 s. At the start of the adaptive sequence, the initial listening level was set to be below the threshold of audibility. This was increased in steps of 10 dB until the subject indicated that the carriers (and increment) were clearly audible, at which point the adaptive procedure began. Three consecutive correct identifications of a ramp resulted in a reduction in the listening level and one incorrect answer resulted in an increase. After each trial, subjects were provided correct/incorrect feedback on their responses. Following a reversal, the step size (starting value of 10 dB) was divided by two. A reversal was defined as an increase in listening level following a decrease, or *vice versa*. After 12 reversals, threshold level was taken as the arithmetic mean of the last 10 reversals. Trials were undertaken in blocks lasting no longer than 20 minutes. Blocks were occasionally interrupted with a break period of 15 minutes, after which the block continued until either the next rest period or completion. Blocks and carrier-frequency orders within blocks were chosen at random. Prior to the test, each subject was provided with a brief demonstration to familiarize themselves with the interface and procedure. A training period was then undertaken which was terminated when the performance of the subject was judged to have stabilized. The data from the training period were not included in subsequent analyses.

## Results

### Modulation filter tuning is carrier-frequency dependent

In order to characterize the tuning of the modulation filter, sensitivity measures must be obtained for two main properties – modulation rate (i.e., rate of change) and modulation depth (i.e., contrast). In the first experiment, we assessed the ability of listeners to detect a change in sound intensity (from a reference intensity), where the change constituted an increment of a defined duration, quantified by the ‘half-ramp’ duration – i.e. the duration from the start of the ramp to its peak. As all ramps were symmetric in time around their peaks, changing the duration of the ramp provides for a proxy of different modulation rates, i.e. faster ramps represent faster modulation rates and slower ramps represent slower rates. Effective modulation depth was held constant at 3dB, so that threshold levels were obtained by assessing the ability of listeners to detect a 3-dB increment for effective modulation rates of 0.5, 5, 10 or 33 Hz for pure tones spanning the range 62 Hz to 16 kHz, i.e., encompassing much of the frequency range of normal-hearing listeners. Absolute sound level was adaptively varied according to the criteria described in the Methods until ~80% performance was reached.

**Figure 2:**
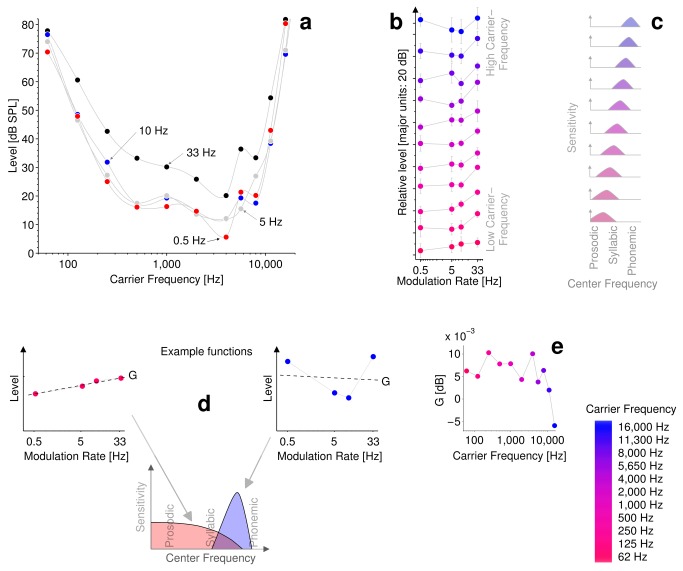
Modulation rate sensitivity. **a** plots group mean threshold-levels as a function of carrier frequency for the nine subjects, for increments of 3 dB at effective modulation rates of 0.5, 5, 10 or 33 Hz. Each data point corresponds to the mean absolute sound-level at which 80% performance was reached for 3-dB ramps of the respective durations. **b** plots the same data as in panel **a** in the form of *modulation-rate-sensitivity functions*, where the data are normalized to remove the effect of absolute threshold. Color scale from red to blue indicates low-to-high carrier frequency. Error bars indicate 95% confidence intervals. *Modulation-rate-sensitivity* functions become increasingly non-monotonic with increase in carrier frequency, indicating a smooth transition in modulation tuning from near-prosodic to near-phonemic rates along the tonotopic gradient. **c** shows an interpretation of the data in terms of corresponding illustrative modulation filter tuning functions, highlighting the variation in modulation filter center-frequency**. d** illustrates our interpretation of G for two example *modulation-rate-sensitivity* functions; large, positive values of G at low carrier-frequencies, indicating modulation filters to be most sensitive to slow (i.e., near-prosodic) modulations. At high carrier-frequencies there is a smaller (even negative) value of G, meaning that the modulation filters are most sensitive to faster (i.e., near-phonemic) modulations. **e** plots G as a function of carrier-frequency.

Figure 2a plots group mean threshold-levels as a function of carrier frequency for the nine subjects, for increments of 3 dB at effective modulation rates of 0.5, 5, 10 or 33 Hz. Each data point corresponds to the mean absolute sound-level at which 80% performance was reached for 3 dB ramps of the respective modulation rate. The overall shape of these curves (equivalent to equal loudness-level contours e.g., see 34) is not greatly affected by ramp duration. However, the overall distance between the functions is smallest at the extremes of the carrier frequency range.

Figure 2b plots the same data as in Figure 2a, but here as *modulation-rate-sensitivity functions*, where the data are normalized to remove the effect of absolute threshold. The main effect of half-ramp duration was verified to be significant in all *modulation-rate-sensitivity* functions (*p* < 0.05, *Friedman Signed-Rank test*), with the exception of those for 62 Hz and 16 kHz. This is likely explained by the combined inter and intra-subject variability associated with extremes of carrier frequency and of half-ramp duration.

The *modulation-rate-sensitivity* function is monotonic for low carrier-frequencies, and non-monotonic (U-shaped) for high carrier-frequencies. The monotonic nature of the functions at low carrier-frequencies is consistent with data from several contemporary studies (e.g., [24–26]) suggesting that increments (or decrements) in sound intensity are detectable in terms of a change in energy (with no reference to the rate of change), whilst the non-monotonic *modulation-rate-sensitivity* functions at high carrier-frequencies are consistent with data reported for similar ramps conveyed in noise [27], which suggest that increment detection might be determined, at least in part, in terms of a change in stimulus contrast. A gradual transition from monotonic functions at low carrier-frequencies to non-monotonic functions at higher carrier-frequencies is evident in the data, with a transition point around 4 kHz. This is in agreement with the findings of Watson and Gengel [20], who demonstrated a faster integration time constant (*τ*) with increasing carrier frequency. However, in both cases, it seems likely that non-monotonic functions would be observed given longer durations on the order of minutes such as those employed by Simpson and Reiss [27].

A critical feature of our method is that the different durations of intensity ramp act as a proxy for modulation rate; short ramps correspond to fast rates and long ramps to slow. By measuring the listening level at which the 80% performance was achieved for the various effective modulation rates, we obtained a measure of the sensitivity of the modulation filter to modulation at each effective rate, i.e. a *modulation-rate-sensitivity* function. A monotonic function implies increasing sensitivity to decreasing rates. A non-monotonic function implies that peak sensitivity is within the range of rates tested. The center frequency of the modulation filter corresponds to the modulation rate at which it is most sensitive. By measuring the regression slope (G) of each *modulation-rate-sensitivity* function, we obtained a measure of how well our range of modulation rates captured the center frequency tuning of the modulation filter at a given carrier frequency. This provides a crude proxy to center frequency tuning. It should be noted that G does not quantify a curve fit to the *modulation-rate-sensitivity* function, but rather is a means of quantifying how well the peak of the modulation filter is centrally captured by the function, i.e., G is informative as to how well the modulation rates represented by each filter are arrayed around the tuned peak. Thus, G = 0 indicates a modulation filter tuned to a carrier frequency at the center of the function, G < 0 indicates a filter tuned to the right of the function’s center and G > 0 a filter tuned to the left of the function’s center.

Figure 2c shows an interpretation of the data in terms of illustrative modulation-filters, corresponding to the *modulation-rate-sensitivity* functions, which illustrate variation in modulation filter center-frequency. This is further illustrated in Figure 2d, for two example *modulation-rate-sensitivity* functions; at low carrier-frequencies there is a large, positive value of G, meaning that modulation filters are most sensitive to slow (i.e., near-prosodic) modulations. At high carrier-frequencies there is a smaller (even negative) value of G, meaning that the modulation filters are most sensitive to faster (i.e., near-phonemic) modulations. Figure 2e plots G as a function of carrier frequency. The steady fall of G with increase in carrier frequency confirms the trend for increasingly high-rate tuned modulation filters along the tonotopic gradient. The narrower dynamic range over which 80% performance was achieved at the extremes of the tonotopic gradient indicates these modulation filters to be relatively broadly tuned, whilst the wider dynamic range at the mid-to-high carrier-frequency end indicates these modulation filters to be more selective for modulation rate.

The data plotted in Figure 2 can be summarized as follows; At low carrier-frequencies, modulation filters appear to be most sensitive to modulation rates that are near-prosodic (i.e. ~1-5 Hz), but towards higher carrier-frequencies the filters appear to be more sensitive to near-phonemic (~20 Hz) modulation rates. Within the range of modulation rates represented in our data, at low carrier-frequencies the modulation filters appear to be low pass and at higher carrier-frequencies the filters appear to be pass band. However, our data do not preclude the possibility that, if slower modulation rates were represented in the function, pass band tuning might be observed for low carrier-frequencies.

### Modulation filter tuning is listening-level dependent

In the second experiment, modulation rate (half-ramp duration) was held constant and the effective modulation depth varied by varying the height of the ramp, to produce a measure of *modulation-depth-sensitivity*. These data were then assessed with respect to sensitivity to modulation rate from the first experiment.

**Figure 3:**
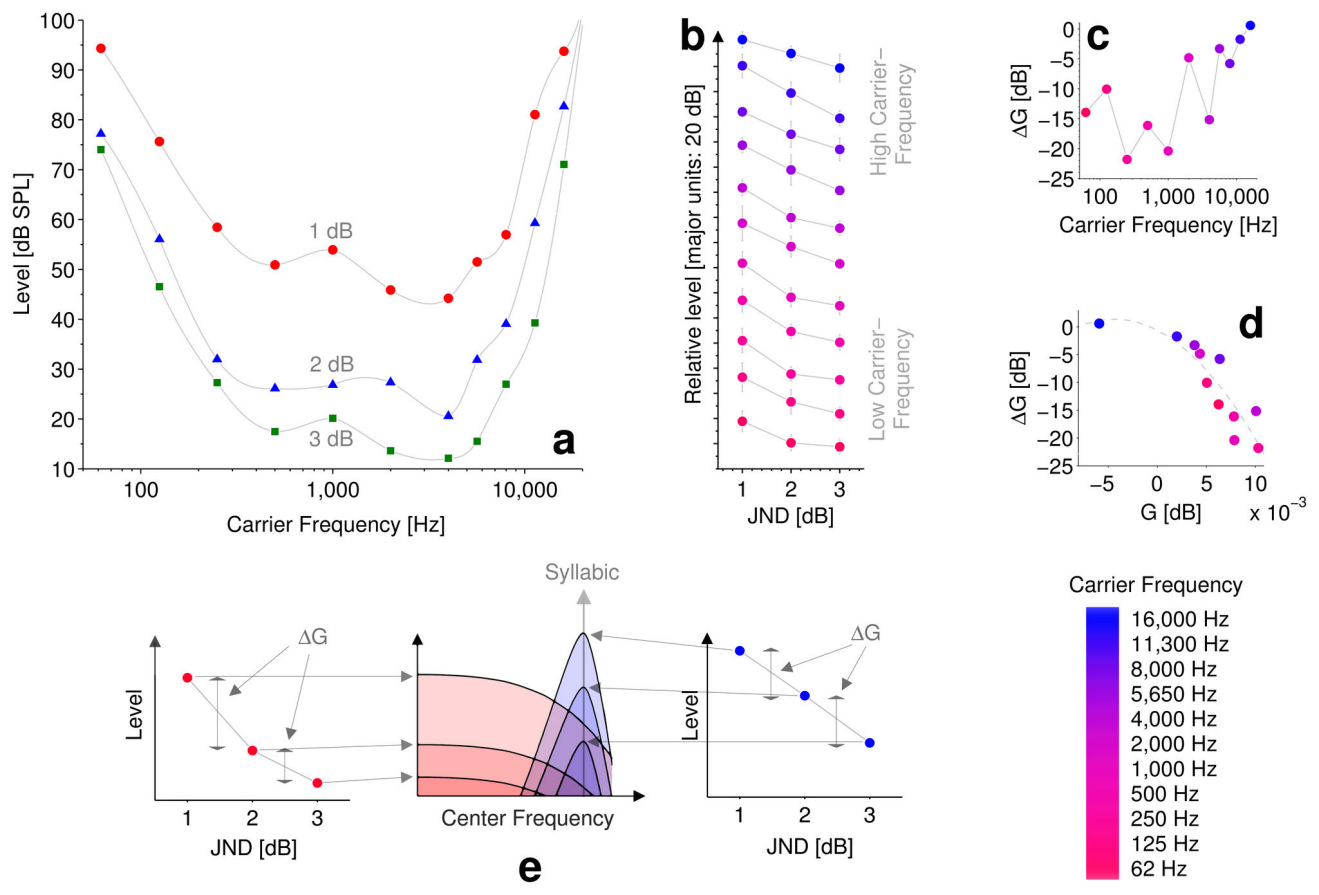
Modulation depth sensitivity. **a** plots contours showing group mean threshold levels at each carrier frequency for the nine subjects, for increments of 1 (red circles), 2 (blue triangles) or 3 (green squares) dB at an effective modulation rate of 5 Hz (i.e., syllabic). Each data point corresponds to the group mean absolute sound-level at which 80% performance was reached for 1, 2 or 3 dB ramps respectively. **b** plots the same data, normalized to produce *modulation-depth-sensitivity* functions. The error bars represent 95% confidence intervals. Color scale (right) from red to blue indicates low-to-high (apical to basal) carrier frequency. **c** plots ΔG as a function of carrier frequency. **d** plots ΔG as a function of G (a proxy to modulation filter center frequency), including a quadratic fit to the data (dashed line) – the two functions are highly correlated (*r* = -0.945, *p* < 5x10^-7^, *Spearman, two-tailed*), thus providing a cross validation for both proxy measures of tuning. **e** shows an interpretation of the data in terms of a cartoon illustration of the interpretation of ΔG for two example *modulation-depth-sensitivity* functions; Near absolute threshold (i.e., for JNDs of 3dB) peripheral spread of modulation plays little role, meaning that coding of the modulation at a given carrier-frequency is dependent only on the modulation filter at the tonotopic location of the carrier-frequency. However, for smaller JNDs level is increased and peripheral spread of the carrier causes spread-of-modulation. Spread of modulation causes the recruitment of modulation filters that are more or less sensitive to syllabic (5 Hz) modulation. For high frequency carriers (blue), the basal modulation filter is most sensitive to the syllabic (5Hz) modulation, and so recruitment of less sensitive filters (by peripheral spread of modulation) has little influence on performance. However, for low-frequency carriers (red), the apical modulation filter is insensitive to the modulation and so at high levels (i.e., 1 dB JND) performance is *enhanced* by more sensitive (basal) modulation filters recruited at basal end of the tonotopic gradient. This enhancement falls away as level is reduced and hence produces the curved functions seen towards apical end of the tonotopic gradient. Small values of ΔG (i.e., at high carrier-frequencies) indicate little effect of spread-of-modulation and large values of ΔG (i.e., at low carrier-frequencies) indicate spread-of-modulation effects.

Figure 3a plots contours showing group mean threshold levels at each carrier frequency for the nine subjects, for increments of 1, 2 and 3 dB at effective modulation rates of 5 Hz. Each data point corresponds to the mean absolute sound-level at which 80% performance was reached for 1, 2 or 3 dB ramps, respectively. In general, the contours of both experiments (Figures 2a & 3a) resemble equal loudness contours, and hence it is reasonable to assume that a major factor in their shape is the outer- and middle-ear transfer function. This is supported by a correlation between Glasberg and Moore’s [34] combined outer-and-middle ear filter and the average contour from all the data of the *temporal and magnitude* experiments (*r* = 0.96, *p* = 3.6x10^-6^, *Pearson, two-tailed*).

The contours of the data in Figure 3a are not parallel, but are most widely spaced in the middle of the carrier-frequency range, and the overall dynamic range of the functions is again smallest at the extremes of the carrier-frequency range. This indicates that *modulation-depth-sensitivity* varies with level most steeply in the middle of the carrier-frequency range. Figure 3b removes (by normalization) the effects of the absolute threshold, allowing the form of the functions to be compared directly. The curved functions at low carrier-frequencies are comparable to the equivalent functions previously reported (e.g., [28]), i.e. the results of previous studies most likely reflect the tuning of the relevant modulation filter at a particular carrier frequency and level. The error bars in Figure 3b represent 95% confidence intervals. Main effect of JND size was verified to be significant in all functions (*p* < 0.05, *Friedman Signed-Rank test*), with the exception of the *modulation-depth-sensitivity* function at 62 Hz. As previously, this is likely a result of the combined inter and intra-subject variability associated with extremes of carrier frequency and of increment size. At high carrier-frequencies, the functions are almost perfectly linear (log-log axes) and so could be predicted with a power law. There is a general trend towards power-law type functions as carrier frequency increases, with a transition after 4 kHz. Furthermore, by comparing the data for 62 Hz and 16 kHz with nearly identical absolute threshold levels at 1 dB (Figure 3a), differences in the shapes of the functions between low and high carrier-frequencies are most apparent. The same comparison is also evident for 125 Hz and 11.3 kHz.

At high carrier-frequencies, as the JND is increased (Figure 3b) the listening level (at threshold) is reduced proportionally. This suggests that tuning is relatively invariant to sound level. However, at low carrier-frequencies, as the JND is increased the listening level (at threshold) is not reduced proportionally, suggesting that tuning changes with sound level. In order to assess the relative changes in tuning of modulation filters at different listening levels, gradients for the level functions of Figure 3b were calculated. For each function, ΔG was calculated as the change in slope between threshold levels for increments of [1,2] dB and [2,3] dB (where a zero value of ΔG indicates power-law type functions). Figure 3c plots ΔG as a function of carrier frequency and shows a steady rise of ΔG with increase in carrier frequency. Figure 3d plots ΔG as a function of G (a proxy to modulation filter center frequency), including a quadratic fit to the data (dashed line) – the two functions are highly correlated (*r* = -0.945, *p* < 5x10^-7^, *Spearman, two-tailed*).

One way of explaining the trends shown in Figure 3c and 3d might be the spread-of-modulation that would result from tonotopically organized CMFs. Figure 3e shows a cartoon illustration of this interpretation of ΔG for two example *modulation-depth-sensitivity* functions; near absolute threshold (i.e., for JNDs of 3dB) peripheral spread of modulation plays little role, meaning that coding of the syllabic (5 Hz) modulation at a given carrier frequency is dependent only on the modulation filter located on the tonotopic gradient according to carrier frequency. However, for smaller JNDs level is increased and peripheral spread of the carrier causes spread-of-modulation. Spread of modulation causes the recruitment of modulation filters that are more or less sensitive to syllabic (5 Hz) modulation. For high frequency carriers (blue), the basal modulation filter is most sensitive to the syllabic (5 Hz) modulation, and so recruitment of less sensitive filters (by peripheral spread of modulation) has little influence on performance. However, for low-frequency carriers (red), the apical modulation filter is insensitive to the syllabic (5 Hz) modulation and so at high levels (i.e., 1 dB JND) performance is *enhanced* by more sensitive modulation filters recruited towards the basal end of the tonotopic gradient. This enhancement falls away as level is reduced and hence produces the curved functions seen towards the apical end of the tonotopic gradient. Therefore, small values of ΔG (i.e., at high carrier-frequencies) indicate little effect of spread-of-modulation and large values of ΔG (i.e., at low carrier-frequencies) indicate spread-of-modulation effects. Following this interpretation, the steady rise of ΔG with increase in carrier frequency indicates a trend describing steady decrease in spread-of-modulation effects across the tonotopic gradient. The correlation shown in Figure 3d provides both a cross validation for both proxy measures of modulation filter tuning, and support for our interpretation of an interaction between modulation-filter tuning and peripheral spread-of-modulation effects. However, it should be noted that spread of modulation is not the only mechanism that may be invoked to explain ΔG - rather it is a mechanism we would expect to see evidence of, based on the suggested cortical tonotopy, and hence is the most plausible interpretation given the correlation with G. Alternative explanations for ΔG might include input/output nonlinearities which are carrier-frequency dependent or CMF bandwidths which change with sound level in a carrier-frequency dependent way.

## Summary

In this study we have provided evidence that human modulation filter tuning is both carrier frequency and level dependent. Our data suggest that CMFs are tonotopic and that the human auditory system is optimized to track rapid (phonemic) modulations at high carrier-frequencies and slow (prosodic) modulations at low carrier-frequencies. We have suggested, based on evidence of modulation filter level dependence, that peripheral spread of excitation is likely to result in ‘spread of modulation’ by spread-of-carrier between CMFs. Furthermore, our data suggests systematic (tonotopic) variation in underlying cortical neuronal physiology. Our data and conclusions provide support for the cortical speech processing strategy suggested by Ding and Simon [13] and confirmation in humans of the findings of Lakatos et al. [17] in monkey CMFs. Carrier-frequency and level-dependent tuning of CMFs may have implications for the cocktail party problem and appear consistent with the ‘dual stream’ hemispheric model suggested in music neuroimaging studies [18].

## References

[1] Humphries C, Liebenthal E, Binder JR (2010) Tonotopic organization of human auditory cortex. Neuroimage 50: 1202-1211. doi:10.1016/j.neuroimage.2010.01.046. PubMed: 20096790. 20096790 10.1016/j.neuroimage.2010.01.046 PMC2830355

[2] Sadagopan S, Wang X (2008) Level invariant representation of sounds by populations of neurons in primary auditory cortex. J Neurosci 28: 3415-3426. doi:10.1523/JNEUROSCI.2743-07.2008. PubMed: 18367608. 18367608 10.1523/JNEUROSCI.2743-07.2008 PMC6670591

[3] Barbour DL (2011) Intensity-invariant coding in the auditory system. Neurosci Biobehav Rev 35: 2064-2072. doi:10.1016/j.neubiorev.2011.04.009. PubMed: 21540053. 21540053 10.1016/j.neubiorev.2011.04.009 PMC3165138

[4] Voss RF, Clarke J (1975) 1/F noise in music and speech. Nature 258: 317-318. doi:10.1038/258317a0.

[5] Voss RF, Clarke J (1978) 1/F noise in music: Music from 1/F noise. J Acoust Soc Am 63: 258-263. doi:10.1121/1.381721.

[6] Garcia-Lazaro JA, Ahmed B, Schnupp JWH (2006) Tuning to natural stimulus dynamics in primary auditory cortex. Curr Biol 16: 264-271. doi:10.1016/j.cub.2005.12.013. PubMed: 16461279. 16461279 10.1016/j.cub.2005.12.013

[7] Garcia-Lazaro JA, Ahmed B, Schnupp JWH (2011) Emergence of Tuning to Natural Stimulus Statistics along the Central Auditory Pathway. PLOS ONE 6(8): e22584. doi:10.1371/journal.pone.0022584. PubMed: 21850231. 21850231 10.1371/journal.pone.0022584 PMC3151266

[8] Wang Y, Ding N, Ahmar N, Xiang J, Poeppel D et al. (2012) Sensitivity to temporal modulation rate and spectral bandwidth in the human auditory system: MEG evidence. J Neurophysiol 107: 2033-2041. doi:10.1152/jn.00310.2011. PubMed: 21975451. 21975451 10.1152/jn.00310.2011 PMC3331605

[9] Dau T, Kollmeier B, Kohlrausch A (1997) Modeling auditory processing of amplitude modulation. I. Detection and masking with narrow-band carriers. J Acoust Soc Am 102: 2892-2905. doi:10.1121/1.420344. PubMed: 9373976. 9373976 10.1121/1.420344

[10] Dau T, Kollmeier B, Kohlrausch A (1997) Modeling auditory processing of amplitude modulation. II. Spectral and temporal integration. J Acoust Soc Am 102: 2906-2919. doi:10.1121/1.420345. PubMed: 9373977. 9373977 10.1121/1.420345

[11] Jepsen ML, Ewert SD, Dau T (2008) A computational model of human auditory signal processing and perception. J Acoust Soc Am 124: 422-438. doi:10.1121/1.2924135. PubMed: 18646987. 18646987 10.1121/1.2924135

[12] Xiang J, Poeppel D, Simon JZ (2013) Physiological evidence for auditory modulation filterbanks: Cortical responses to concurrent modulations. J Acoust Soc Am 133: EL7 PubMed: 23298020. 23298020 10.1121/1.4769400 PMC3555506

[13] Ding N, Simon JZ (2013) Adaptive temporal encoding leads to a background-insensitive cortical representation of speech. J Neurosci 33: 5728-5735. doi:10.1523/JNEUROSCI.5297-12.2013. PubMed: 23536086. 23536086 10.1523/JNEUROSCI.5297-12.2013 PMC3643795

[14] Shannon RV, Zeng FG, Kamath V, Wygonski J, Ekelid M (1995) Speech recognition with primarily temporal cues. Science 270: 303-304. doi:10.1126/science.270.5234.303. PubMed: 7569981. 7569981 10.1126/science.270.5234.303

[15] Drullman R, Festen JM, Plomp R (1994) Effect of reducing slow temporal modulations on speech reception. J Acoust Soc Am 95: 2670-2680. doi:10.1121/1.409836. PubMed: 8207140. 8207140 10.1121/1.409836

[16] Zion Golumbic EM, Ding N, Bickel S, Lakatos P, Schevon CA et al. (2013) Mechanisms underlying selective neuronal tracking of attended speech at a “cocktail party”. Neuron 77: 980-991. doi:10.1016/j.neuron.2012.12.037. PubMed: 23473326. 23473326 10.1016/j.neuron.2012.12.037 PMC3891478

[17] Lakatos P, Musacchia G, O’Connel MN, Falchier AY, Javitt DC et al. (2013) The spectrotemporal filter mechanism of auditory selective attention. Neuron 77: 750-761. doi:10.1016/j.neuron.2012.11.034. PubMed: 23439126. 23439126 10.1016/j.neuron.2012.11.034 PMC3583016

[18] Zatorre RJ, Zarate JM (2012) Cortical processing of music. In: Poeppel D Overath T Popper AN Fay RR The human auditory cortex, springer handbook of auditory research. Berlin: Springer pp. 261-294.

[19] Brungart DS, Chang PS, Simpson BD, Wang D (2006) Isolating the energetic component of speech-on-speech masking with ideal time-frequency segregation. J Acoust Soc Am 120: 4007-4018. doi:10.1121/1.2363929. PubMed: 17225427. 17225427 10.1121/1.2363929

[20] Watson CS, Gengel RW (1969) Signal duration and signal frequency in relation to auditory sensitivity. J Acoust Soc Am 46: 989-997. doi:10.1121/1.1911819. PubMed: 5824042. 5824042 10.1121/1.1911819

[21] Jesteadt W, Wier CG, Green DM (1977) Intensity discrimination as a function of frequency and sensation level. J Acoust Soc Am 61: 169-177. doi:10.1121/1.381278. PubMed: 833368. 833368 10.1121/1.381278

[22] Long GR, Cullen JK (1985) Intensity difference limens at high frequencies. J Acoust Soc Am 78: 507-513. doi:10.1121/1.392472. PubMed: 4031249. 4031249 10.1121/1.392472

[23] Ozimek E, Zwislocki JJ (1996) Relationships of intensity discrimination to sensation and loudness levels: Dependence on sound frequency. J Acoust Soc Am 100: 3304-3320. doi:10.1121/1.416993. PubMed: 8914312. 8914312 10.1121/1.416993

[24] Oxenham AJ (1997) Increment and decrement detection in sinusoids as a measure of temporal resolution. J Acoust Soc Am 102: 1779-1790. doi:10.1086/231161. PubMed: 9301055. 9301055 10.1121/1.420086

[25] Plack CJ, Gallun FJ, Hafter ER, Raimond A (2006) The detection of increments and decrements is not facilitated by abrupt onsets or offsets. J Acoust Soc Am 119: 3950-3959. doi:10.1121/1.2198184. PubMed: 16838538. 16838538 10.1121/1.2198184

[26] Gallun FJ, Hafter ER (2006) Amplitude modulation sensitivity as a mechanism for increment detection. J Acoust Soc Am 119: 3919-3930. doi:10.1121/1.2200136. PubMed: 16838535. 16838535 10.1121/1.2200136

[27] Simpson AJR, Reiss JD (2013) The Dynamic Range Paradox: A Central Auditory Model of Intensity Change Detection. PLOS ONE 8(2): e57497. doi:10.1371/journal.pone.0057497. PubMed: 23536749. 23536749 10.1371/journal.pone.0057497 PMC3585315

[28] Viemeister NF, Bacon SP (1988) Intensity discrimination, increment detection and magnitude estimation for 1-kHz Tones. J Acoust Soc Am 84: 172-178. doi:10.1121/1.2025969. PubMed: 3411045. 3411045 10.1121/1.396961

[29] Weber EH (1846) Der Tastsinn und das Gemeingefühl. In: Wagner R Handwörterbuch der Physiologie mit Rücksicht auf physiologische Pathologie 3. Braunschweig: Friedr. Vieweg & Sohn pp. 481-588.

[30] Miller GA (1947) Sensitivity to changes in the intensity of white noise and its relation to masking and loudness. J Acoust Soc Am 19: 609-619. doi:10.1121/1.1916528.

[31] McGill W, Goldberg J (1968) A study of the near-miss involving Weber’s law and pure tone intensity discrimination. Percept Psychophys 4: 105-109. doi:10.3758/BF03209518.

[32] Peters RW, Moore BCJ, Glasberg BR (1995) Effects of level and frequency on the detection of decrements and increments in sinusoids. J Acoust Soc Am 97: 3791-3799. doi:10.1121/1.412394. PubMed: 7790657. 7790657 10.1121/1.412394

[33] Levitt H (1971) Transformed up-down methods in psychoacoustics. J Acoust Soc Am 49: 467-477. doi:10.1121/1.1912375. PubMed: 5541744. 5541744

[34] Moore BCJ, Glasberg BR, Baer T (1997) A model for the prediction of thresholds, loudness, and partial loudness. J Audio Eng Soc 45: 224-240.

